# 

**Published:** 1858-02

**Authors:** 


☞ The Medical press of the United States is respectfully requested to copy the foregoing.

